# Supplementary material for: Unprecedented UK heatwave harmonised drivers of fuel moisture creating extreme temperate wildfire risk
Source: Commun Earth Environ. 2025 Sep 1;6(1):727. doi: 10.1038/s43247-025-02746-8 (PMC12401727; doi:10.1038/s43247-025-02746-8)
Supplement: Supplementary file 2 — Supplementary Information [file 43247_2025_2746_MOESM2_ESM.pdf]

Table S1: Landscape characteristics of the sample sites within each region and the range in temperature/humidity during the July 2022 heatwave (Scottish Highlands were not sampled during the heatwave). Soil types include a brief descriptor of soil type and the corresponding Soilscapes classification[1] (for full description of soil types see Table S7). Land cover types: AG = acid grassland, CF = coniferous forest, H = heathland, B = bog.

**Region**	**Soil types**	**Elevation range (m)**	**Land cover types**	**Heatwave temperature range (°C)**	**Heatwave humidity range (%)**
Scottish Highlands	Peat (19, 25)	47 - 70	H	N/A	N/A
North York Moors	Sandy (14), peat/peat surface (16, 19, 25)	175 - 415	CF, H, B	29.2 - 36.7	31.0 - 46.0
Peak District	Sandy (14), peat/peat surface (16, 25)	366 - 504	AG, CF, H, B	26.8 - 37.0	23.9 - 47.7
East Anglia	Chalky (3), sandy (10, 11), peat surface (23)	9 - 42	AG, CF, H	33.3 - 42.8	15.5 - 25.6
Southeast	Sandy (14, 15), loamy (22)	35 - 63	CF, H	32.6 - 40.3	18.7 - 39.0
Southwest/South Wales	Loamy/sandy (6, 14), peat surface (16)	229 - 541	AG, CF, H	23.5 - 29.3	39.6 - 56

Table S2: Mean FMC (%) of live *Calluna* canopy (LC), dead *Calluna* canopy (DC) and the organic layer (O) at different time periods within each region. Separate numbers beside each group of regions denote overall mean.

Fuel layer	Region of UK	Spring 2021		July 2021		Spring 2022		July 2022 (heatwave)	
**LC**	East Anglia	66.8	74.0	133.0	128.3	49.6	68.8	84.3	107.8
	North York Moors	90.0		127.8		66.3		132.5	
	Peak District	69.0		106.5		67.8		116.5	
	South East	68.7		133.8		81.3		79.3	
	South Wales/South West	75.4		140.3		79.1		126.5	
**DC**	East Anglia	13.1	16.8	10.4	17.7	13.9	19.0	3.8	4.2
	North York Moors	27.6		32.5		21.0		3.2	
	Peak District	9.9		9.8		14.3		4.2	
	South East	13.1		20.3		18.3		3.7	
	South Wales/South West	20.4		15.5		27.6		6.0	
**O**	East Anglia	95.7	238.0	26.9	148.0	28.4	156.0	5.4	78.4
	North York Moors	N/A		173.4		184.0		85.2	
	Peak District	304.1		172.5		237.4		113.4	
	South East	98.1		67.6		86.4		26.7	
	South Wales/South West	286.2		179.0		193.3		124.2	

Table S3: Wilcoxon tests comparing the FMC of live *Calluna* canopy, dead *Calluna* canopy and organic layer for spring 2021, July 2021, spring 2022 and 17th- 19th July 2022 (dates of the heatwave). W = smaller of rank totals. Models with significant p-values (i.e. FMC of compared time periods are significantly different; <0.05) are shaded in bold.

	Live *Calluna* canopy		Dead *Calluna* canopy		Organic layer	
Test	W	p-value	W	p-value	W	p-value
Spring 2021 vs Spring 2022	722	0.113	666	0.054	**835**	**<0.001**
Spring 2021 vs July 2021	**120**	**<0.001**	853	0.214	**546**	**<0.001**
Spring 2022 vs July 2021	**87**	**<0.001**	862	0.080	972	0.951
Spring 2021 vs July 2022 (heatwave)	**172**	**<0.001**	**1287**	**<0.001**	**611**	**<0.001**
Spring 2022 vs July 2022 (heatwave)	**172**	**<0.001**	**1225**	**<0.001**	**1359**	**<0.001**
July 2021 vs July 2022 (heatwave)	**760**	**0.002**	**1016**	**<0.001**	**891**	**<0.001**

Table S4: Fire Behaviour Predictions and Probability of Ignition for spring (average of April 2021 & April 2022), July 2021 and July 2022 heatwave in the South East of the UK. Model input variables (dead/live fuel moisture content and temperature) are also shown.

Time Period	**Input**: Dead Fuel Moisture (%)	**Input**: Live Fuel Moisture (%)	**Input**: Temperature (°C)	**Output**: Surface Fire Rate of Spread (m/min)	**Output**: Flame Length (m)	**Output**: Probability of Ignition (%)
Mean Spring (April 2021 & 2022)	15.7	75	11	10.1	1.2	11
Mean July 2021	20.3	133.8	21	5.2	0.9	5
Heatwave July	3.7	79.3	42	22.5	2	87

Table S5: Nearest weather stations with available MIDAS Open weather data[2] and the Environment Information Data Centre (EIDC) weather data[3] to each region of the UK where sample sites were located. Data were downloaded from each weather station to predict missing temperature data for sample sites.

**Region of UK**	**Weather station**
Scottish Highlands	Forsinard Flows RSPB Reserve (EIDC)
North York Moors	Fylingdales (MIDAS)
Peak District	Sheffield (MIDAS)
East Anglia	Santon Downham (MIDAS)
South Wales	Libanus (MIDAS)
South West	Liscombe (MIDAS)
South East	Alice Holt Lodge (MIDAS)

Table S6: Soil categories associated with sample sites taken from Soilscapes[1].

Soilscapes category	Soilscapes description	Soil grouping
3	Shallow lime-rich soils over chalk or limestone	Loamy & freely draining
6	Freely draining slightly acid loamy soils	Loamy & freely draining
10	Freely draining slightly acid sandy soils	Sandy & freely draining
11	Freely draining sandy Beckland soils	Sandy & freely draining
14	Freely draining very acid sandy and loamy soils	Sandy & freely draining
15	Naturally wet very acid sandy and loamy soils	Sandy & naturally wet
16	Very acid loamy upland soils with a wet peaty surface	Peaty & naturally wet
19	Slowly permeable wet very acid upland soils with a peaty surface	Peaty & naturally wet
22	Loamy soils with a naturally high groundwater	Loamy & naturally wet
23	Loamy and sandy soils with naturally high groundwater and a peaty surface	Peaty & naturally wet
25	Blanket bog peat soils	Peaty & naturally wet

Table S7: Two-stage model summaries. Weather model investigates the effect of weather and phenological variables on organic fuel moisture content (FMC), using site as a random effect. Landscape model investigates the effect of each landscape variable on the effect size of site from weather model.

Organic weather model			*Marginal R2 = 0.134*	*Conditional R2 = 0.699*
term	estimate	se	statistic	*p* value
intercept	0.106	0.123	0.859	0.395
VPD	-0.131	0.033	-4.016	0.000
long-term daily air temperature	-0.181	0.032	-5.663	0.000
days since rainfall	0.016	0.027	0.606	0.545
total five day rainfall	0.060	0.028	2.175	0.030
NDVI	0.018	0.026	0.697	0.486
time of day	0.018	0.024	0.732	0.464
sample year (2022)	-0.418	0.058	-7.241	0.000
sample year (2023)	-0.362	0.117	-3.090	0.002
Organic landscape model			*adj. R2=0.605*	
term	estimate	se	statistic	*p* value
intercept	-0.572	0.236	-2.421	0.021
land cover (bog)	0.158	0.353	0.447	0.658
land cover (coniferous forest)	0.296	0.221	1.342	0.189
land cover (heathland)	0.200	0.216	0.923	0.363
elevation	0.661	0.131	5.051	0.000
slope	-0.097	0.125	-0.779	0.442
aspect	-0.063	0.089	-0.702	0.488
soil (loamy naturally wet)	1.376	0.550	2.500	0.018
soil (peaty naturally wet)	0.478	0.248	1.923	0.063
soil (sandy freely-draining)	0.137	0.245	0.558	0.581
soil (sandy naturally wet)	0.849	0.323	2.630	0.013

Table S8: Two-stage model summaries. Weather model investigates the effect of weather and phenological variables on live heather canopy fuel moisture content (FMC), using site as a random effect. Landscape model investigates the effect of each landscape variable on the effect size of site from weather model.

Live canopy weather model			*Marginal R2 = 0.229*	*Conditional R2 = 0.318*
term	estimate	se	statistic	*p* value
intercept	0.003	0.036	0.077	0.939
VPD	-0.071	0.025	-2.879	0.004
long-term daily air temperature	0.165	0.025	6.625	0.000
days since rainfall	-0.038	0.020	-1.882	0.061
total five day rainfall	0.034	0.021	1.634	0.103
NDVI	0.064	0.020	3.203	0.001
time of day	-0.038	0.017	-2.200	0.028
sample year (2022)	-0.012	0.043	-0.278	0.781
sample year (2023)	-0.081	0.086	-0.943	0.346
Live canopy landscape model			*adj. R2=0.370*	
term	estimate	se	statistic	*p* value
intercept	-0.070	0.051	-1.356	0.194
land cover (bog)	-0.002	0.061	-0.033	0.974
land cover (heathland)	-0.035	0.041	-0.860	0.403
elevation	0.024	0.024	0.973	0.345
slope	0.009	0.024	0.365	0.720
aspect	0.011	0.018	0.611	0.550
soil (peaty naturally wet)	0.123	0.054	2.258	0.038
soil (sandy freely-draining)	0.081	0.057	1.435	0.171
soil (sandy naturally wet)	0.060	0.064	0.944	0.359

Table S9: Summary of weather model which investigates the effect of weather and phenological variables on dead heather canopy fuel moisture content (FMC), using site as a random effect. The variance in FMC explained by sites was close to zero (marginal R2 is equal to conditional R2), indicating that FMC does not vary depending on site (and therefore landscape variables), so the landscape model was not carried out for this fuel type.

Dead canopy weather model			*Marginal R2 = 0.471*	*Conditional R2 = 0.471*
term	estimate	se	statistic	*p* value
intercept	-1.738	0.048	-36.270	0.000
VPD	-0.457	0.047	-9.726	0.000
long-term daily air temperature	-0.054	0.047	-1.151	0.251
days since rainfall	-0.032	0.040	-0.808	0.420
total five day rainfall	0.145	0.040	3.658	0.000
NDVI	0.021	0.038	0.539	0.590
time of day	-0.131	0.034	-3.830	0.000
sample year (2022)	-0.160	0.082	-1.952	0.052
sample year (2023)	-0.018	0.240	-0.074	0.941

Table S10: The correlation between each variable within the weather/phenology models. Significant correlations ($p < 0.05$) are shown in bold. VPD = Vapour Pressure Deficit, LMDT = long-term mean daily temperature, NDR = number of days since rain, 5DR = five day rainfall, TD = time of day.

Organic

	VPD	LMDT	NDVI	NDR	5DR	TD
VPD	**1.00**					
LMDT	**0.55**	**1.00**				
NDVI	**0.08**	**0.35**	**1.00**			
NDR	**0.30**	**0.10**	0.02	**1.00**		
5DR	**-0.26**	**-0.09**	**-0.15**	**-0.52**	**1.00**	
TD	**0.10**	**0.17**	**0.11**	-0.07	**-0.01**	**1.00**

Live heather canopy

	VPD	LMDT	NDVI	NDR	5DR	TD
VPD	**1.00**					
LMDT	**0.56**	**1.00**				
NDVI	**0.18**	**0.47**	**1.00**			
NDR	**0.29**	**0.12**	0.07	**1.00**		
5DR	**-0.26**	-0.07	**-0.17**	**-0.50**	**1.00**	
TD	0.07	**0.13**	**0.10**	-0.06	0.04	**1.00**

Dead heather canopy

	VPD	LMDT	NDVI	NDR	5DR	TD
VPD	**1.00**					
LMDT	**0.54**	**1.00**				
NDVI	**0.18**	**0.45**	**1.00**			
NDR	**0.29**	**0.11**	0.05	**1.00**		
5DR	**-0.27**	-0.09	**-0.17**	**-0.49**	**1.00**	
TD	0.06	0.12	**0.10**	**-0.11**	**0.07**	**1.00**

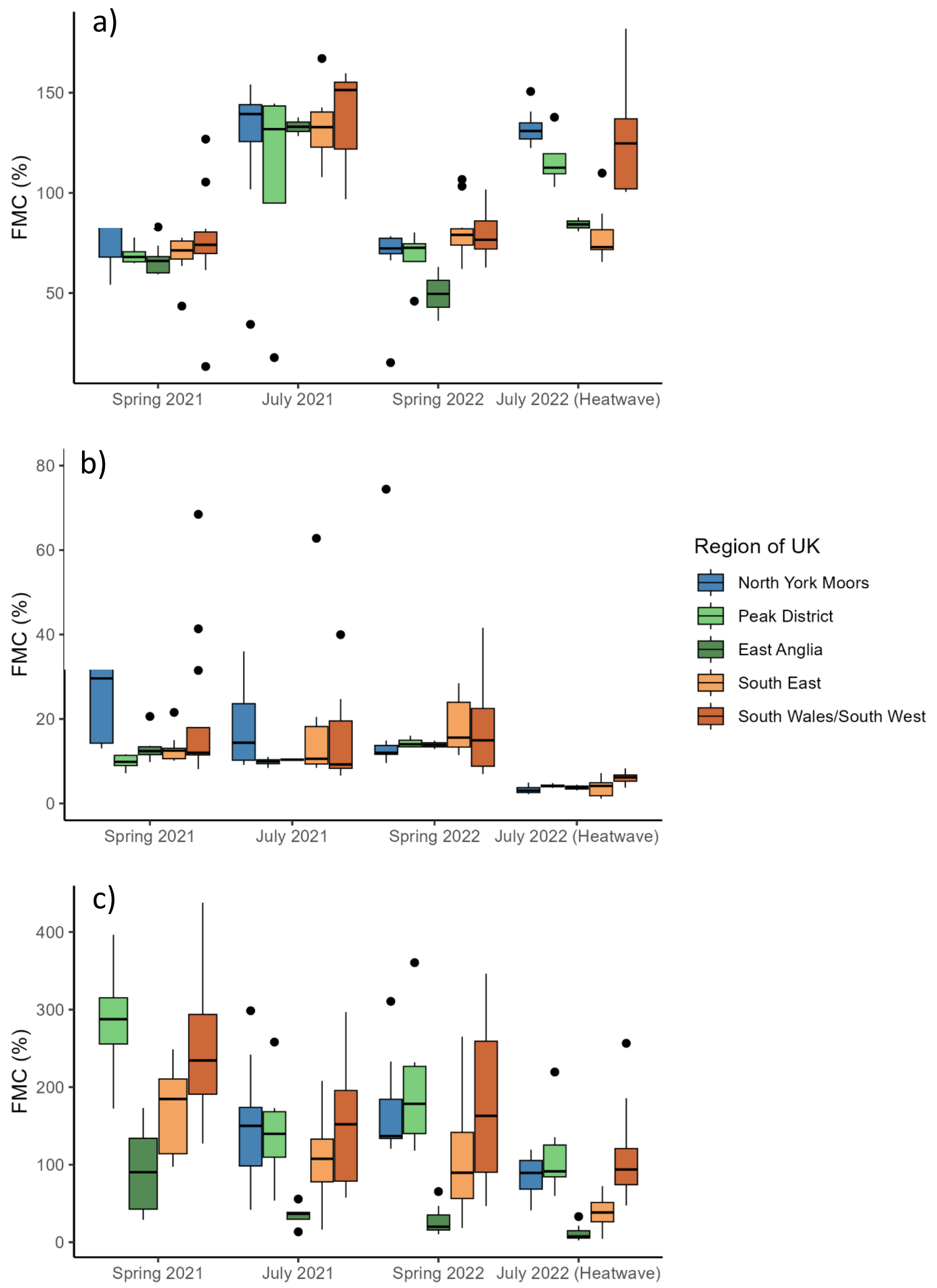

Figure S1: Moisture content of (a) live Calluna canopy, (b) dead Calluna canopy and (c) the organic soil layer during Spring (March-April) 2021, July 2021, Spring (March-April) 2022 and the heatwave of 2022 (17th-19th July) from sample sites in different regions of the UK.

Organic layer

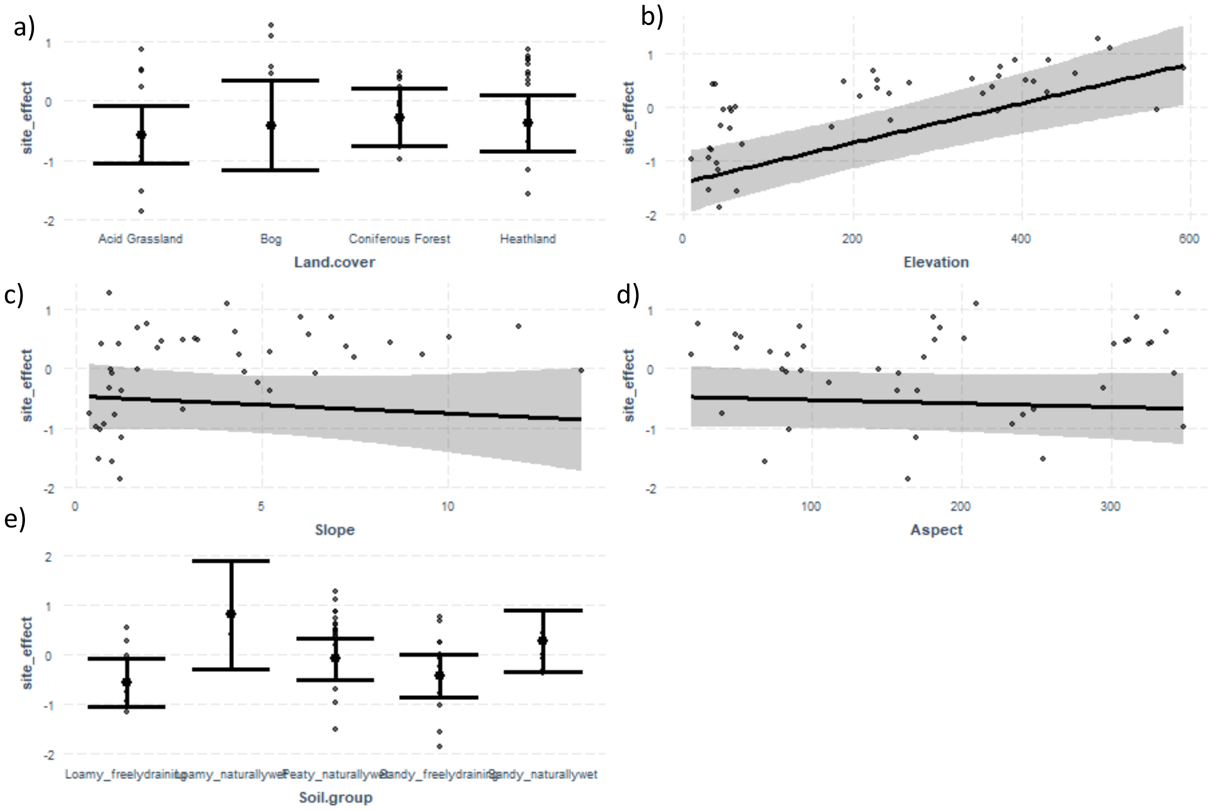

Figure S2: Partial effect plots showing the relationship between across-site variation of FMC of the organic layer and landscape factors associated with sample sites (a = land cover type; b = elevation (metres); c = slope (degrees); d = aspect (degrees); e = soil group (classified using Soilscapes (Farewell et al., 2011)). Model presented was the second model in a two-stage modelling process; the first mixed-effects linear model compared FMC with weather and phenological variables, using sample site as a random effect. The effect sizes for sample site were extracted and used in a second linear model which compared these effect sizes to landscape variables.

Live heather canopy

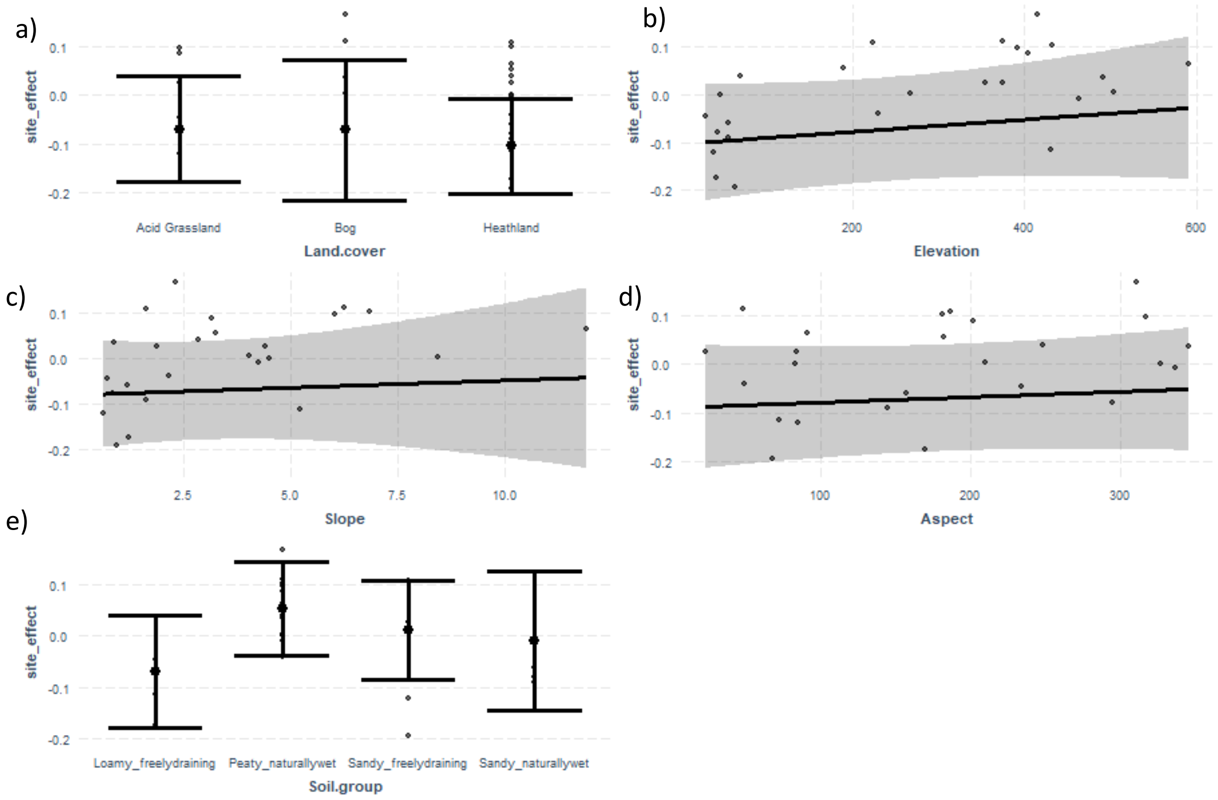

Figure S3: Partial effect plots showing the relationship between across-site variation of FMC of live heather canopy and landscape factors associated with sample sites (a = land cover type; b = elevation (metres); c = slope (degrees); d = aspect (degrees); e = soil group (classified using Soilscapes (Farewell et al., 2011)). Model presented was the second model in a two-stage modelling process; the first mixed-effects linear model compared FMC with weather and phenological variables, using sample site as a random effect. The effect sizes for sample site were extracted and used in a second linear model which compared these effect sizes to landscape variables.

Table S8: Dates on which fuel moisture content was sampled, fuel types sampled on each date and the associated regions and land cover types. **Region**: SE = South East, SW = South Wales/South West, EA = East Anglia, PD = Peak District, NYM = North York Moors, SH = Scottish Highlands. **Land cover types:** H = heathland, AG = acid grassland, B = bog, C = coniferous forest.

Date	Region	Land cover types
24/02/2021	SE	H
03/03/2021	SW	H, AG, C
09/03/2021	SE	H, C
12/03/2021	NYM	B
15/03/2021	EA	AG, H
17/03/2021	SW	H, AG, C
24/03/2021	SE	H, C
26/03/2021	NYM	B, H
29/03/2021	EA	AG, H
31/03/2021	SW	H, AG, C
12/04/2021	EA	AG, H
14/04/2021	SW	H, AG, C
16/04/2021	PD	H, AG, B, C
21/04/2021	SE	H, C
23/04/2021	PD	B, C
24/04/2021	PD	AG, H, B, C
26/04/2021	EA	AG, C
27/04/2021	SW	H, AG, C
05/05/2021	SE	H, C
07/05/2021	NYM	B, C
10/05/2021	EA	AG, H, C
12/05/2021	SW	H, AG, C
14/05/2021	SW	H, AG, C
18/05/2021	PD	B, AG, H, C
19/05/2021	SE	H, C
24/05/2021	EA	AG, H, C
27/05/2021	NYM	H, B, C
03/06/2021	NYM	H, B
07/06/2021	EA	AG, H, C
09/06/2021	SW	AG, H, C
11/06/2021	SW	AG, H, C
14/06/2021	PD	B, AG, H, C
16/06/2021	SE	H, C
23/06/2021	SW	H, AG, C
28/06/2021	PD	B, AG, H, C
30/06/2021	SE	H, C
02/07/2021	NYM	B, H, C
05/07/2021	EA	AG, H, C
09/07/2021	SW	H, AG, C
14/07/2021	SE	H, C
16/07/2021	NYM	H, B, C
21/07/2021	SW	H, AG, C
22/07/2021	SW	H, AG, C
26/07/2021	PD	AG, H, B, C
28/07/2021	SE	H, C
30/07/2021	NYM	B, H, C
02/08/2021	EA	AG, H, C
04/08/2021	SW	H, AG, C
06/08/2021	SW	AG, H, C

10/08/2021	PD	B, AG, H, C
11/08/2021	SE	H, C
12/08/2021	NYM	B, H, C
16/08/2021	EA	AG, H, C
17/08/2021	SW	AG, H, C
20/08/2021	SW	AG, H, C
23/08/2021	PD	B, AG, H, C
25/08/2021	SE	H, C
27/08/2021	NYM	B, H, C
01/09/2021	SW	H, AG, C
08/09/2021	SE	H, C
20/09/2021	SW	AG, H
22/09/2021	NYM	B, H
30/09/2021	SW	C
08/10/2021	SE	H, C
11/10/2021	EA	AG, H, C
12/10/2021	NYM	B, H, C
19/10/2021	PD	H, AG, C
21/10/2021	SE	H, C
26/10/2021	NYM	B, H, C
01/11/2021	EA	AG, H, C
03/11/2021	SW	H, AG, C
11/11/2021	SW	AG, H, C
15/11/2021	PD	B, AG, H, C
18/11/2021	SE	H, C
22/11/2021	NYM	H, C
25/11/2021	SW	H, AG, C
29/11/2021	EA	AG, C
02/12/2021	SW	AG, H, C
10/12/2021	SE	H, C
31/01/2022	SE	H, C
01/02/2022	NYM	B, H, C
08/02/2022	PD	AG, H, C
10/02/2022	SW	AG, H, C
14/02/2022	SE	H, C
06/03/2022	SW	AG, H, C
07/03/2022	SW	H, AG, C
08/03/2022	NYM	H, C
21/03/2022	SE	H, C
23/03/2022	SW	AG, H
24/03/2022	SW	AG, H, C
25/03/2022	NYM	B, H, C
26/03/2022	SE	H, C
25/04/2022	EA	AG, H, C
26/04/2022	SW	AG, H, C
27/04/2022	SW	H, AG, C
28/04/2022	PD	AG, B, H, C
29/04/2022	SE	H, C
09/05/2022	EA	AG, H, C
17/05/2022	SE	H, C
19/05/2022	SW	AG, H
25/05/2022	SH	H
26/05/2022	SH	H
16/06/2022	NYM	B, H, C

17/06/2022	PD, SE	B, AG, H, C
21/06/2022	SW	AG, H, C
23/06/2022	SW	AG, H, C
30/06/2022	SH	H
11/07/2022	EA	AG, H, C
12/07/2022	SE	H, C
13/07/2022	PD	B, AG, H, C
17/07/2022	SW	AG, H, C
18/07/2022	EA, NYM, SE	AG, H, B, C
19/07/2022	NYM, PD, SE	B, H, AG, C
21/07/2022	SH	H
07/08/2022	NYM, PD	H, B, C
09/08/2022	EA	AG, H, C
10/08/2022	SE	H, C
11/08/2022	SW	AG, H, C
30/08/2022	SW	AG, H, C
31/08/2022	SW	AG, H, C
01/09/2022	PD	B, AG, H, C
19/09/2022	SE	H, C
20/09/2022	SW	AG, H, C
04/02/2023	NYM, PD, SW, NYM	H, AG, B, C
18/02/2023	SW	AG, H, C
19/02/2023	SE , PD	H, B, C
22/04/2023	SW, NYM	AG, H, B, C
30/05/2023	SW, PD, NYM, SE	H, AG, B, C
04/06/2023	NYM , NYM, PD	H, B, C
05/06/2023	PD, SW, NYM	H, AG, B, C

References:

1. Farewell, T. S., Truckell, I. G., Keay, C. A. & Hallett, S. H. The use and applications of the Soilscapes datasets. *Cranfield University* (2011).
2. Met Office. Met Office MIDAS Open: UK Land Surface Stations Data (1853-current). *Centre for Environmental Data Analysis, 14.6.23* (2019).
3. Coyle, M. *et al.* Carbon dioxide and methane fluxes and associated environmental observations from an unmodified blanket bog, Forsinard Flows RSPB Reserve, Scotland, 2016-2022. *NERC EDS Environmental Information Data Centre.* (2024).
